# Transcriptome Profiling to the Effects of Drought Stress on Different Propagation Modes of Tea Plant (*Camellia sinensis*)

**DOI:** 10.3389/fgene.2022.907026

**Published:** 2022-08-10

**Authors:** Zhou Ding, Changjun Jiang

**Affiliations:** ^1^ School of Tea and Food Science Technology, Anhui Agricultural University, Hefei, China; ^2^ State Key Laboratory of Tea Biology and Utilization, Anhui Agricultural University, Hefei, China

**Keywords:** transcriptome, sexually progeny, asexual reproduction, drought stress, *Camellia sinensis* (L.)O.Ktze

## Abstract

Tea plant (*Camellia sinensis*) is an important economic beverage crop. Drought stress seriously affects the growth and development of tea plant and the accumulation of metabolites, as well as the production, processing, yield and quality of tea. Therefore, it is necessary to understand the reaction mechanism of tea plant under drought conditions and find efficient control methods. Based on transcriptome sequencing technology, this study studied the difference of metabolic level between sexual and asexual tea plants under drought stress. In this study, there were multiple levels of up-regulation and down-regulation of differential genes related to cell composition, molecular function and biological processes. Transcriptomic data show that the metabolism of tea plants with different propagation modes of QC and ZZ is different under drought conditions. In the expression difference statistics, it can be seen that the differential genes of QC are significantly more than ZZ; GO enrichment analysis also found that although differential genes in biological process are mainly enriched in the three pathways of metabolic, single organism process and cellular process, cellular component is mainly enriched in cell, cell part, membrane, and molecular function, and binding, catalytic activity, and transporter activity; the enrichment order of differential genes in these pathways is different in QC and ZZ. This difference is caused by the way of reproduction. The further study of these differential genes will lay a foundation for the cultivation methods and biotechnology breeding to improve the quality of tea.

## Introduction

Tea (*Camellia sinensis* (L.) Kuntze, Theaceae) is an important economic plant worldwide (Das et al., 2015), which has high economic value (Xue et al., 2013; Lou et al., 2015; Yang et al., 2016): the leaves of it are long round or oval, which can be used to make tea (Kessenich and Higgs, 2010), a very popular drink (Hodgson et al., 2002; Hodgson et al., 2005); the seeds can be used for oil (Mantil et al., 2015; Morsoleto et al., 2015; Tai et al., 2015); the tree is good materials and it can be used for carving. There are two modes of propagation of tea plants: sexual (seed raised) and clonal (derived from stem cutting), mainly clonal propagation (Zhang et al., 2021).

Drought is among the most severe constraint of all biotic and abiotic stresses, thereby limiting crop productivity of dryland farming and threatening world food security (Nouraei et al., 2022). Tea plant is an evergreen plant that likes warm temperature and humidity, and its drought tolerance is weak (Dong et al., 2017; Guo et al., 2017). Because of the global warming, drought is becoming more and more frequent, which has become one of the most serious natural disasters and is also an important factor restricting the growth of tea plants (Liu et al., 2016a; Li et al., 2019b; Li J. et al., 2019). Severe drought and high temperature will cause the transformation of tea leaves from dry to dead (Liu et al., 2016a; Zheng et al., 2016; Rahimi, Kordrostami and Mortezavi, 2019; Xie et al., 2019). Phenotypic symptom analysis showed that drought reduced the water content of tea plants and the weight of buds and roots (Wei et al., 2010; Jin-Long et al., 2012; Wu et al., 2019). The changes in these indicators indicate that tea plants work hard to cope with drought in some ways, but drought still hindering the growth and metabolism of tea plants at all stages, affecting the structure of tea plants while reducing the quality and yield of tea leaves (Xue et al., 2013; Tai et al., 2015; Zheng et al., 2016; Guo et al., 2017).

Over the past decades, great progress has been made in explaining the genetic and molecular basis of drought responses in plants such as *Arabidopsis* (Zhang P. et al., 2019). Drought also promotes the production of reactive oxygen species (ROS) that can damage cells. To survive drought, plants have developed enzymatic and non-enzymatic antioxidative defense mechanisms to scavenge ROS and ROS scavenging enzymes activities like superoxide dismutase (SOD), guaiacol peroxidase (POD), ascorbate peroxidase (APX), and catalase (CAT) (Zhang P. et al., 2019; Ansari et al., 2022). The physiological mechanism of tea under drought stress has also been partially studied in recent years (Jin et al., 2021; Zhang et al., 2021), The results showed that drought stress would hinder the growth and metabolism of tea plants and increase the content of soluble protein and proline. (Liu et al., 2016a; Liu et al., 2016b), The activity of SOD and other antioxidant enzymes significantly improved the ability of ROS scavenging mechanism, because the content of ROS in plants caused by drought exceeds the range of scavenging ability under the original equilibrium state (Sen and Alikamanoglu, 2012). In addition, drought reduced the photosynthesis of tea plants, resulting in water loss of stomatal cells, decreased stomatal conductance, and CO_2_ entering stomata; thus, the photosynthetic rate (Chen et al., 2011). ABA accumulation of tea plants will increase under drought stress, which is conducive to maintaining a higher water state of plants, thus reducing stress injury (Shinozaki and Yamaguchi-Shinozaki, 1992; Yoshida et al., 2010; Sreenivasulu et al., 2012; Bai et al., 2019; Song et al., 2019). At the same time, drought can change the contents of metabolites such as total polyphenols, total catechins, free amino acids and caffeine in tea plants, which has a great influence on the quality of tea (Li et al., 2020).

However, at present, the research of tea drought stress mainly uses clonal cultivated species as materials (Zhang et al., 2021). It is not clear whether there are differences between sexual lines and clones under drought stress. With the rapid development of second-generation sequencing technology (Ertl et al., 2011; Kaur et al., 2011; Wang et al., 2012), transcriptome sequencing has been completed in more and more plants, and a large number of transcriptome data sequences have been obtained, which saves a lot of work for transcriptome screening and promotes the application in plants (Kim et al., 2014). Compared with model plants such as *Arabidopsis thaliana*, the molecular mechanism of drought tolerance of tea plants is not much (Abo Gamar et al., 2019; Roca Paixao et al., 2019). Drought tolerance phenomenon is a complex trait that involves several metabolic and morphological adaptive pathways. Deciphering genetic basis of drought stress tolerance mechanisms in tea plants still remains a challenging task (Ansari et al., 2022). The application of transcriptome technology to tea plant response to drought stress can be used for secondary metabolic pathways (Kaur et al., 2011; Deng et al., 2016) and regulation research (Covington et al., 2008; Li et al., 2015; Zhao et al., 2017; Li et al., 2019a), resistance research (Kim et al., 2014; Li et al., 2019a; Zhang X.-Y. et al., 2019), functional gene mining, and so on (Niinemets et al., 2018).

In this experiment, the transcriptome of tea tree was sequenced by RNA-Seq sequencing using the drought-prone treatment of the progeny and asexual progeny of the clonal tea tree. The gene annotation was obtained by Blast alignment, followed by Unigene and NR on the tea tree sample. The database was screened to find the molecular mechanism of response to high-temperature and drought stress in different reproductive modes at the level of gene transcription. The differentially expressed genes related to amino acids and enzymes in tea leaves under drought stress, such as proline, were obtained. The key regulatory genes in the POD, SOD, and CAT biosynthesis pathways provide theoretical guidance for analyzing the drought-resistant functional genes of the above tea plants, and lay the foundation for improving the quality of tea plants by improving cultivation methods and biotechnology breeding.

## Materials and Methods

### Plant Materials and Drought Stress Treatments

**FIGURE 1:**
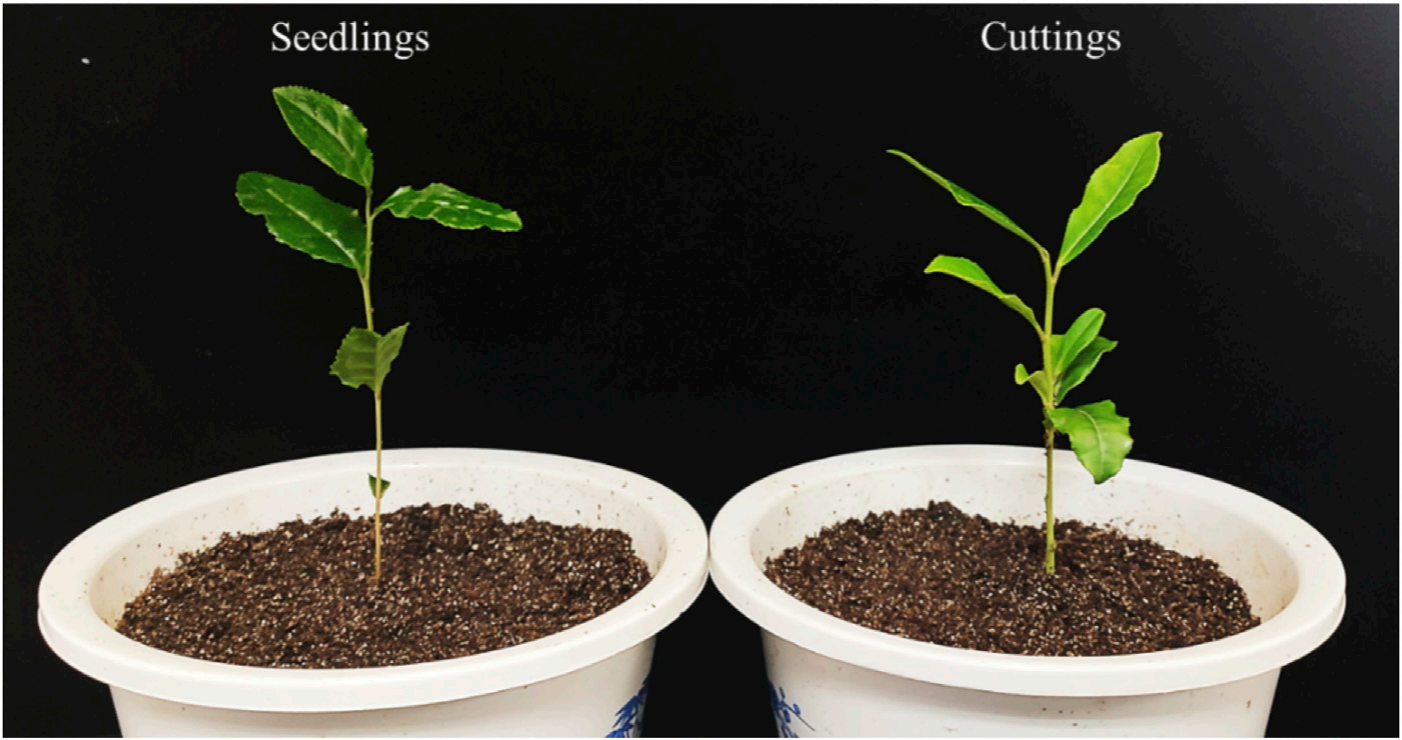
Picture of seedings and cuttings tea plant.

One-year-old tea cultivars, Shuchazao (2n = 2x = 30 chromosomes, *Camellia sinensis* var. assamica (J.W.Mast.) Kitam, Theaceae), were planted in plastic pots (1 plant per pot) at the Hefei, Anhui China Figure 1. 18 pots of cutting seedlings and 18 pots of seed seedlings, respectively. Tea seedlings were uniformly cultured in an artificial climate chamber with a temperature of 25 ± 0.5°C and a humidity of 80%. During drought treatment, the portable soil moisture meter (VICOMETER) is used to detect the soil moisture content and control the soil moisture in each period. The water content of soil was controlled at about 50% on the 5th day, about 40% on the 10th day, about 30% on the 15th day, about 20% on the 20th day, and about 15% on the 25th day. The control treatment was irrigation every day (9:00-10:00 a.m., the irrigation amount was 1.0 L per pot, and the relative water content of soil reached 80%). For physiological experiments, 200 mg roots was harvested and pooled for each treatment group at 0, 5, 10, 15, 20, and 25 days, and the collection was repeated three times as biological replicates. In order to more accurately compare the differences between the two breeding methods under drought stress, we selected the samples treated on the 20th day (moderate drought) for transcriptome sequencing, and 0 day as the control. The cuttings were named QC and the seed seedlings were named ZZ. There were at least three repetitions per process.

### RNA Isolation and Transcriptome Sequencing

The two sets (0 and 20 days) of collected roots were subjected to RNA isolation using the TRIzol reagent in accordance with the manufacturer’s instructions. The quantity and purity of total RNA were assessed using a NanoDrop Photometer Spectrophotometer (IMPLEN, Westlake Village, CA, United States), and 1% agarose gel electrophoresis. Following the examination of quality and quantity, the libraries for transcriptome sequencing ware prepared using Illumina’s kit following the manufacturer recommendations. The RNA libraries were sequenced on the Illumina sequencing platform by Genedenovo Biotechnology Co., Ltd., (Guangzhou, China).

### RNA-Seq Data Analysis

Quality control of the data from RNA-Seq was utilized with SeqPrep (https://github.com/jstjohn/SeqPrep) yichuand Sickle (https://github.com/najoshi/sickle). Adapter and primer sequences were removed, and sequences with lengths below 20  bp were discarded. After removing adapter and primer sequences, low-quality bases were trimmed from the 3′ end of the reads. After trimming low-quality bases, sequences with quality values less than 10 were discarded. Sequences with N ratios higher than 10% were also removed. The error rate (%), Q20 and Q30 values, GC-content (%), and sequence duplication levels of the resulting high-quality clean reads were then evaluated.

Reference genome sequences and gene annotation files were downloaded from TPIA (https://tpia.teaplant.org/index.html) and our RNA-seq reads were aligned to the tea reference genome using TopHat v2.1.0 (Wei et al., 2018). Gene annotation was used to guide read mapping and no more than 2 mismatches were allowed. Reads per kilobase per million reads (RPKMs) were calculated to estimate gene expression levels using HTSeq (version 0.6.1).

For functional annotation and classification, all transcripts and their corresponding genes were compared with the Clusters of Orthologous Groups of proteins (COG, https://www.ncbi.nlm.nih.gov/COG/), Gene Ontology (GO, https://www.geneontology.org) and Kyoto Encyclopedia of Genes and Genomes (KEGG, https://www.genome.jp/kegg/) databases. GO analysis was conducted using the BLAST2GO software with default parameters. COG functional classification was conducted using Blastx software in the STRING database. KEGG pathway annotation was performed using KOBAS (https://kobas.cbi.pku.edu.cn/).

### Differential Gene Expression Analysis

The expression of Unigenes can be calculated by RPKM method, and the gene expression abundance obtained can be directly used to compare the expression differences between different samples. Log2 and FCFDR were used to screen the differentially expressed genes. The screening conditions were |log2FC| > 1 and FDR < 0.05.

### Quantitative RT-PCR Assay

To determine mRNA levels of drought-responsive genes, quantitative real time PCR was performed. The remaining samples of the transcriptome were used to perform qRT-PCR, total RNA was extracted from drought treated and control group, and then reverse transcribed into cDNA. The specific primers were designed by Primer Premier 5.0 software and used to quantify the expression of genes, and actin was used as internal control (Table 1). All qRT-PCR experiments were repeated 3 biological replicates and each biological replicate had 6 technical replicates.

**TABLE 1:** qRT-PCR primers.

Primer name	Sequence 5′-3′
CsGAPDH-F	TTG​GCA​TCG​TTG​AGG​GTC​T
CsGAPDH-R	CAG​TGG​GAA​CAC​GGA​AAG​C
CsActin-F	GCC​ATC​TTT​GAT​TGG​AAT​GG
CsActin-R	GGT​GCC​ACA​ACC​TTG​ATC​TT
QC TEA006283-F	TGA​CTA​ACC​CGC​CAA​CAA​CT
QC TEA006283-R	ACC​AAC​CCG​CCA​AGA​AGA​T
QC TEA018992-F	CAT​ACA​AAT​GCC​AGC​TCC​CA
QC TEA018992-R	TGA​GAG​GGC​CAC​TAG​GTC​GT
QC TEA010590-F	TGC​CAA​TTT​CTC​TGC​TCC​TG
QC TEA010590-R	TCA​AGT​TCC​ACA​CCG​ACG​TT
QC TEA016075-F	CGG​ATC​GTT​GCC​TAG​TTC​AC
QC TEA016075-R	CGC​ATT​CGA​CCT​CTT​CTG​AC
QC TEA004537-F	CAA​ACC​CAC​AAG​CGC​AAG​TA
QC TEA004537-R	TCA​CTG​CCC​AAG​AAT​CGT​TC
QC TEA005361-F	ACC​GCA​TCA​CCA​CTA​CCA​CA
QC TEA005361-R	CAC​TCT​GCC​GAT​CCG​AAA​T
QC TEA008983-F	GGG​TTT​GAC​CTC​GCA​ACT​TC
QC TEA008983-R	GCA​TGA​CAC​GCA​ATA​GGG​AT
QC TEA026818-F	CCC​ATA​CGG​TGA​ATA​CTG​GC
QC TEA026818-R	CCC​TGT​GAT​CGA​CGA​AAT​GT
QC TEA009673-F	TGT​TCT​TCC​ACC​GGG​TTC​C
QC TEA009673-R	AAC​ACC​GGC​CCA​TAT​CTC​TG
QC TEA016430-F	AGA​ATC​CGG​GCT​GCA​TGT​AT
QC TEA016430-R	AGT​CCC​AAG​CCA​GAG​TCG​AT
ZZ TEA015199-F	CGC​ATG​GAC​AAT​GAG​GTG​AT
ZZ TEA015199-R	TTG​CGG​CAC​AAT​ACA​GCT​CT
ZZ TEA010880-F	CGC​CAC​AGT​TGG​AAA​TTC​TG
ZZ TEA010880-R	AAG​CCA​AGA​TTG​GAA​CTC​CC
ZZ TEA026349-F	TAG​CCA​CTG​AAT​GCG​GGA​TA
ZZ TEA026349-R	CAA​TCG​CTG​CTC​TGG​AGT​GT
ZZ TEA006156-F	GGG​GCC​ATC​AAT​GTT​CCT​TA
ZZ TEA006156-R	CAA​GCT​GGC​ATC​CGA​CAA​TA
ZZ TEA015880-F	ACG​AGA​TAG​GGG​TTC​TTG​CC
ZZ TEA015880-R	GAA​TCC​CTT​TCC​TTT​CCA​GC
ZZ TEA008472-F	TTG​GCA​AGT​TCG​ACA​CGT​CT
ZZ TEA008472-R	AAG​CCA​ACC​CTA​GCA​AGC​CT
ZZ TEA004071-F	CCT​TGG​CTT​TGG​CAT​CAG​TA
ZZ TEA004071-R	AAC​ATG​ACC​TTG​GGC​GAC​AT
ZZ TEA001821-F	CAC​ATT​CTC​GTC​CCC​ATG​AA
ZZ TEA001821-R	TGC​TCG​AAG​AGG​TTG​TGG​GT
ZZ TEA004537-F	CAA​ACC​CAC​AAG​CGC​AAG​TA
ZZ TEA004537-R	TCA​CTG​CCC​AAG​AAT​CGT​TC
ZZ TEA016076-F	GCG​CGG​TTT​TCT​CAT​TCC​TA
ZZ TEA016076-R	AAC​CTC​TCG​GGC​ATG​AAT​TG

### Determination of Physiological Indexes Related to Drought Stress

For each tea plant, biomass was measured at the end of drought treatment. The leaves and stems are collected together for aboveground biomass. All roots collected in pot plants were collected to obtain root biomass. The collected samples were then dried at 70°C for 48 h. And then, the dried samples are measured to calculate the biomass. The contents of soluble sugar, soluble protein, proline, malondialdehyde (MDA), superoxide dismutase (SOD), peroxidase (POD), and catalase (CAT) were measured by commercial kit (Suzhou Comin Biotechnology Co., Ltd., China), respectively. All procedures are performed according to the kit instructions.

### Data Analyses

All data were analyzed by SPSS (version 20.0). Data of plant biomass were described using their dry weight, and the root to shoot ratio (R/S) was calculated using the following formula (Peng et al., 2019).

R/S=Broot/Baboveground

where Broot was the root biomass and Baboveground was the aboveground biomass. Values reported here are means of eight replicates. Significance testing was analyzed using Student’s t-test and one-way ANOVA. Significance was considered for *p* < 0.05. **p < 0.01, and ***p < 0.0001. Graphical work was carried out using Origin software 8.0.

## Results

### Physiological Indexes of Sexual and Clonal Tea Plants Under Drought Stress

The effect of drought stress on physiological indicators in seedlings and cuttings of tea plant is shown in Table 2. For seedlings, the soluble sugar content reached the peak at 15d, which was 5.78 mg/g. The lowest soluble sugar content appeared at 5d, about 1.18 mg/g. The soluble sugar content of 15d was significantly higher than that of other groups (*p* < 0.05). The soluble sugar content of 20d was significantly higher than CK, 5d, 10d, and 25d (*p* < 0.05). The soluble sugar content of 10d was significantly higher than CK, 5d, and 25d (*p* < 0.05). The soluble sugar content of 25d was significantly higher than CK and 5d (*p* < 0.05).

**TABLE 2:** Effect of drought stress on physiological indicators in seedlings and cuttings of tea plant.

Materials	Item	Drought stress levels						SEM
		CK	5d	10d	15d	20d	25d	
Seedlings	soluble sugar (mg/g)	1.21^e^	1.18^e^	3.35^c^	5.78^a^	4.25^b^	2.95^d^	0.23
	soluble protein (mg/g)	12.21^d^	13.18^d^	15.35^c^	17.78^b^	19.34^a^	17.95^b^	0.41
	proline (μg/g)	17.08^e^	18.35^e^	21.25^d^	28.59^c^	35.05^b^	38.37^a^	1.21
	MDA (μmol/g)	6.36^e^	6.46^e^	7.35^d^	8.87^c^	10.63^a^	10.17^b^	0.25
Cuttings	soluble sugar (mg/g)	1.35^d^	1.25^d^	2.28^c^	3.95^a^	3.11^b^	2.23^c^	0.14
	soluble protein (mg/g)	12.61^d^	13.65^d^	15.28^c^	17.05^b^	18.68^a^	16.83^b^	0.36
	proline (μg/g)	17.53^d^	18.65^d^	21.36^c^	26.45^ab^	28.96^a^	27.65^a^	0.67
	MDA (μmol/g)	6.26^e^	6.42^e^	7.26^d^	9.82^c^	11.96^b^	13.37^a^	0.41

SEM: standard error of mean.

Means within the same row with different superscripts are significantly different (*p* < 0.05). ^a^, ^b^, ^c^,^d^ indicate that after Duncan test, *p* < 0.05, the difference is significant

The changes of soluble protein and MDA content were similar. The highest content of soluble protein and MDA appeared at 20d, which were 19.34 mg/g and 10.63 μmol/g, respectively. And the lowest points of these two indexes appeared in the CK group, which were 12.21 mg/g and 6.36 μmol/g, respectively. The soluble protein and MDA content of 20d were significantly higher than that of other groups (*p* < 0.05). The soluble protein and MDA content of 25d was significantly higher than CK, 5d, 10d, and 15d (*p* < 0.05). The soluble protein and MDA content of 15d was significantly higher than CK, 5d, and 10d (*p* < 0.05). The soluble protein and MDA content of 10d was significantly higher than CK and 5d (*p* < 0.05).

The proline content gradually increased with increasing drought stress levels. After 25 days of drought stress, it reached the highest point of 38.37 μg/g. Without drought stress, the proline content in leaves was the lowest, which was 17.08 μg/g. The proline content of 25d was significantly higher than that of other groups (*p* < 0.05). The proline content of 20d was significantly higher than CK, 5d, 10d, and 15d (*p* < 0.05). The proline content of 15d was significantly higher than CK, 5d, and 10d (*p* < 0.05). The proline content of 10d was significantly higher than CK and 5d (*p* < 0.05).

For cuttings, the content changes of soluble sugar and soluble protein were similar. The highest contents of soluble sugar and soluble protein appeared at 15d and 20d, respectively. The lowest contents of soluble sugar and soluble protein appeared at 5d and CK, respectively. The soluble sugar content of 20d was significantly higher than CK, 5d, 10d, and 25d (*p* < 0.05). The soluble protein content of 15d was significantly higher than CK, 5d, 10d, and 25d (*p* < 0.05). The soluble sugar content of 10d was significantly higher than CK, 5d, and 25d (*p* < 0.05). The soluble protein content of 25d was significantly higher than CK, 5d, and 10d (*p* < 0.05). However, there was no significant difference in soluble sugar and soluble protein between of CK and 5d.

The content changes of proline and MDA were similar. The proline and MDA content of cuttings gradually increased with increasing drought stress levels. Until 20 days of drought, the concentration of proline reached highest 28.96 μg/g. And until 25 days of drought, the concentration of MDA reached highest 13.37 μmol/g. The lowest contents of proline and MDA were 17.53 μg/g and 6.26 μmol/g without drought stress, respectively. The proline content of 25d was significantly higher than CK, 5d, 10d, and 15d (*p* < 0.05). The MDA content of 20d was significantly higher than CK, 5d, 10d, and 15d (*p* < 0.05). The proline and MDA content of 15d was significantly higher than CK, 5d, and 10d (*p* < 0.05). The proline and MDA content of 10d was significantly higher than CK and 5d (*p* < 0.05). Based on the above indicators, day 0 and day 20 were selected for transcriptome sequencing.

### Overview of Sequencing and Splicing

**FIGURE 2:**
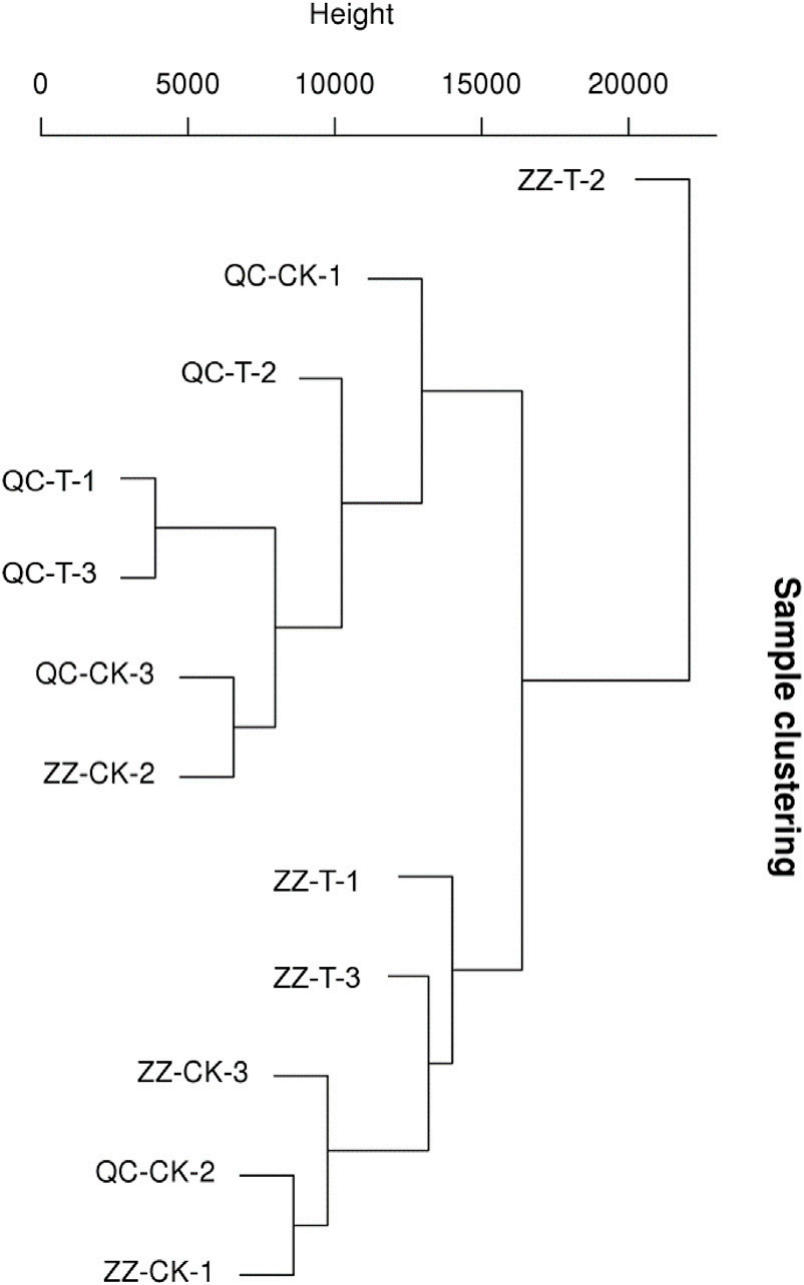
Sample Cluster Diagram. QC, Cutting tea plant. ZZ, Seeding tea plant. CK: Cutting samples treated with drought for 0 days, T: Cutting samples treated with drought for 20 days.

The quality monitoring and filtering results are shown in table 2. The number of clean reads obtained from each sample is about 50 million. In addition, the total number of tested genes was 33,932, the known genes were 30,680, and the new genes were 7,120 (Table 3). As shown in Figure 2, QC has a good correlation, while ZZ has a complicated genetic background due to its sexual reproduction and a slightly higher deviation degree of ZZ-2. The data were uploaded to NCBI with BioProject ID (PRJNA822682).

**TABLE 3:** Statistics of gene expression results.

All Reference Genes	Known Gene Num	New Gene Num
33932	30680 (90.42%)	7120

**FIGURE 3:**
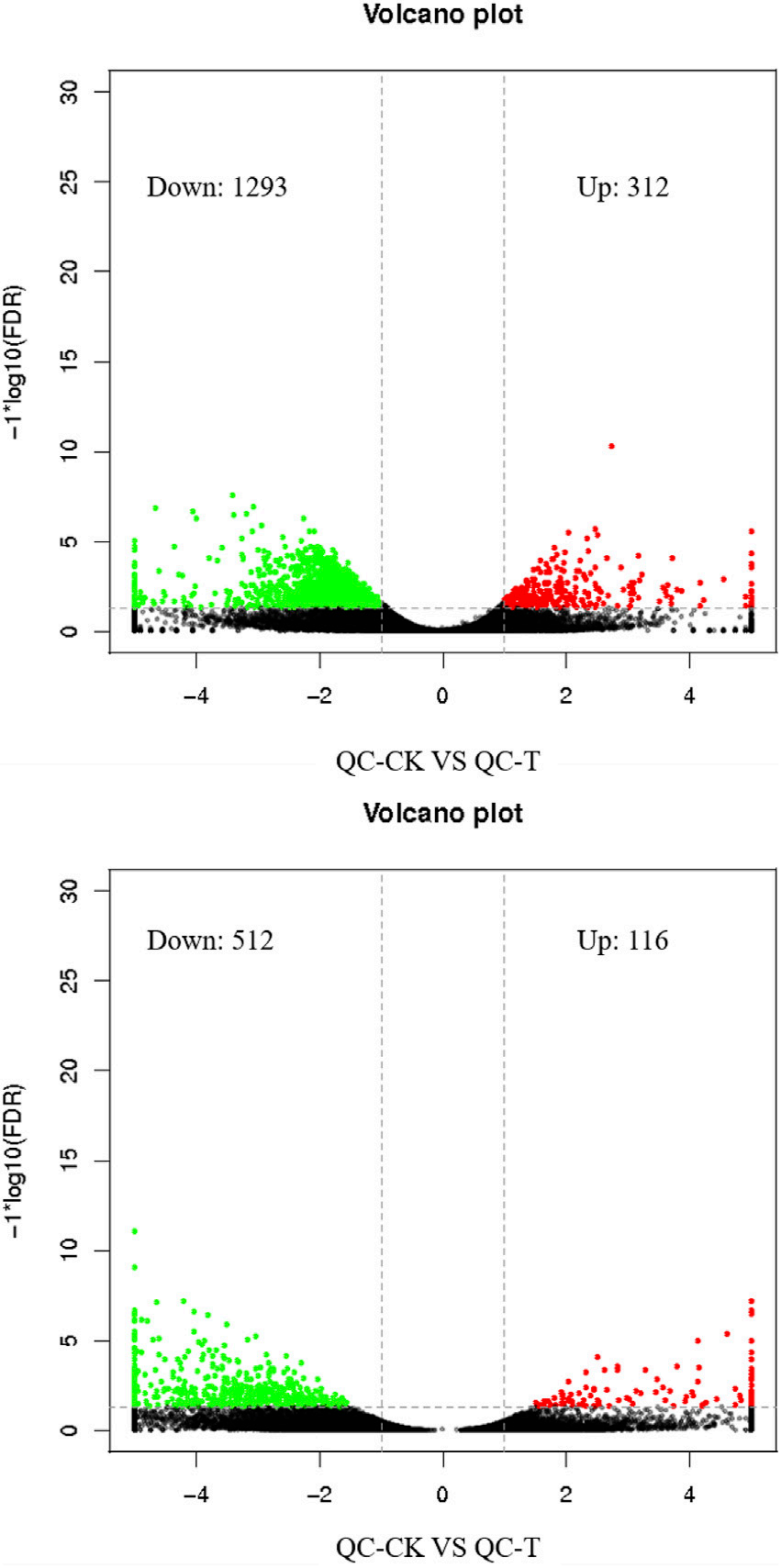
Histogram of differential gene statistics between groups. QC, Cutting tea plant. ZZ, Seeding tea plant. CK: Cutting samples treated with drought for 0 days, T: Cutting samples treated with drought for 20 days.

Through edge R software for gene expression quantity analysis of differences between groups, tea tree cutting propagation of asexual descendants treated with drought stress the differentially expressed genes increase to 312, in the form of cut to 1,293, tea seed reproductive health offspring after drought stress treatment of differentially expressed genes increases for 116, cut the number of 512 (Figure 3).

### Functional Analysis of Transcriptome

**FIGURE 4:**
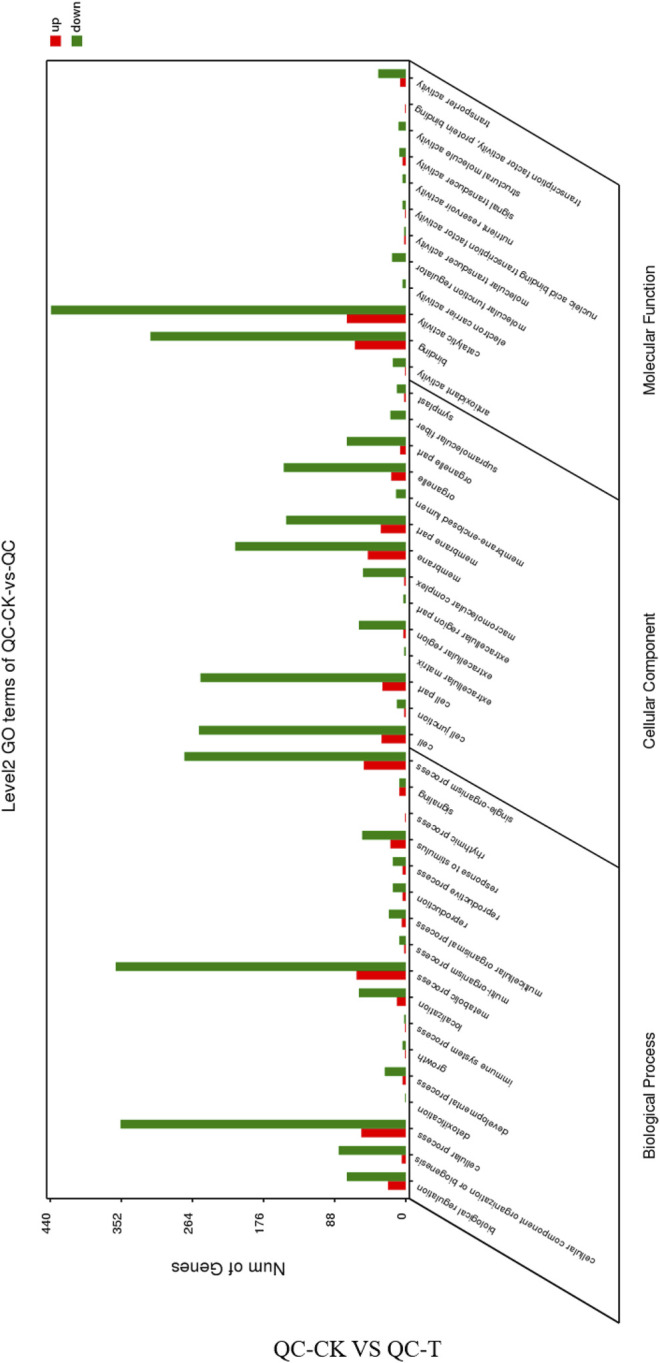
GO function annotation of the differentially expressed genes in QC tea plantation. QC, Cutting tea plant. CK: Cutting samples treated with drought for 0 days, T: Cutting samples treated with drought for 20 days.

GO annotation and functional enrichment analysis were performed on the differentially expressed genes obtained from the drought treatment of the asexual descendants of tea tree cuttings, respectively, in the biology process, cellular component and molecular function. The up-regulated genes were 255, 152, and 171, while the down-regulated genes were 1,356, 853, and 1,262, respectively (Figure 4). The distribution of up-regulated genes and down-regulated genes in GO function is consistent. Biological regulation, cellular component organization or biogenesis, cellular process, localization, metabolic process, response to stimulus, single-organism process, cell, cell part, and extracellular are all involved Significant enrichment is achieved in matrix, membrane part, organelle part, binding, transporter activity, and catalytic activity.

**FIGURE 5:**
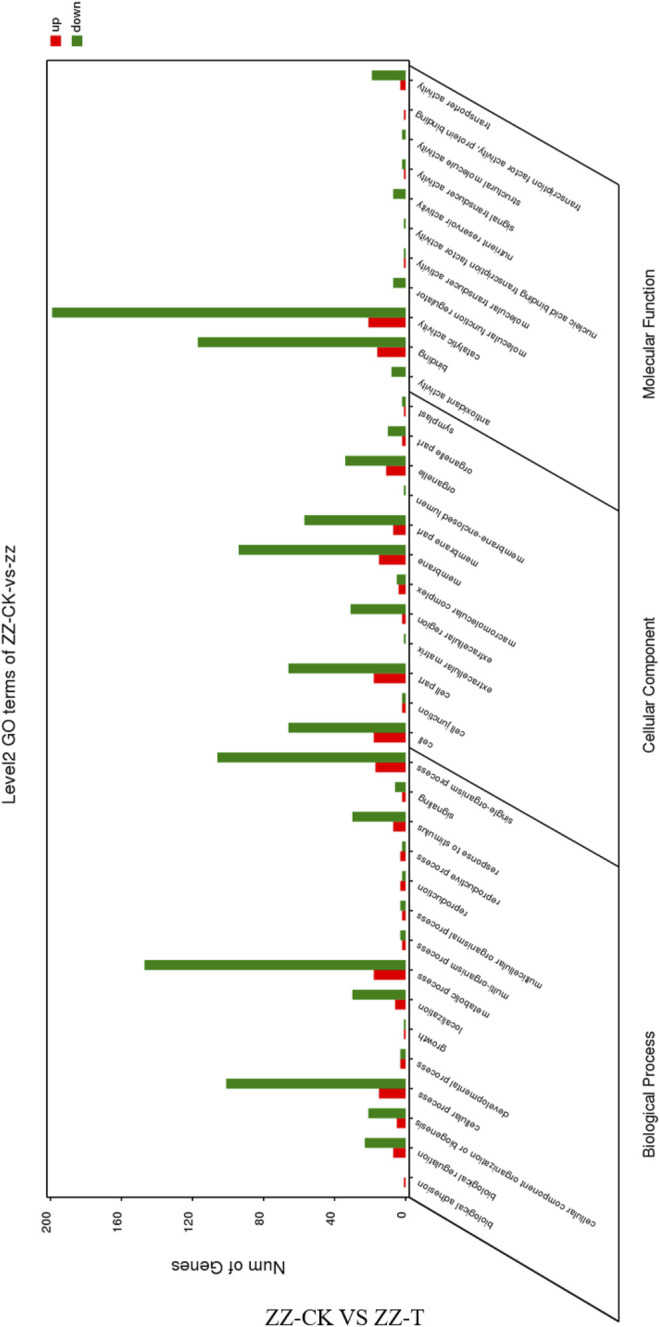
GO function annotation of the differentially expressed genes in ZZ tea plantation. ZZ, Seeding tea plant. CK: Cutting samples treated with drought for 0 days, T: Cutting samples treated with drought for 20 days.

GO annotation and functional enrichment analysis were performed on the differentially expressed genes obtained by drought treatment in the vegetative offspring of tea tree seed propagation. In biological processes, cell components, and molecular functions of the three categories of notes, there are up-down. The up-regulated genes were 92, 43, 80 and down-regulated genes were 47, 363, 369, respectively (Figure 5). Up-regulated genes and down-regulated genes are found in biological regulation, cellular component organization or biogenesis, cellular process, localization, metabolic process, response to stimulus, single-organism process, cell, cell part, and extracellular cells. Significant enrichment is achieved in matrix, membrane part, organelle part, binding, transporter activity and catalytic activity.

**FIGURE 6:**
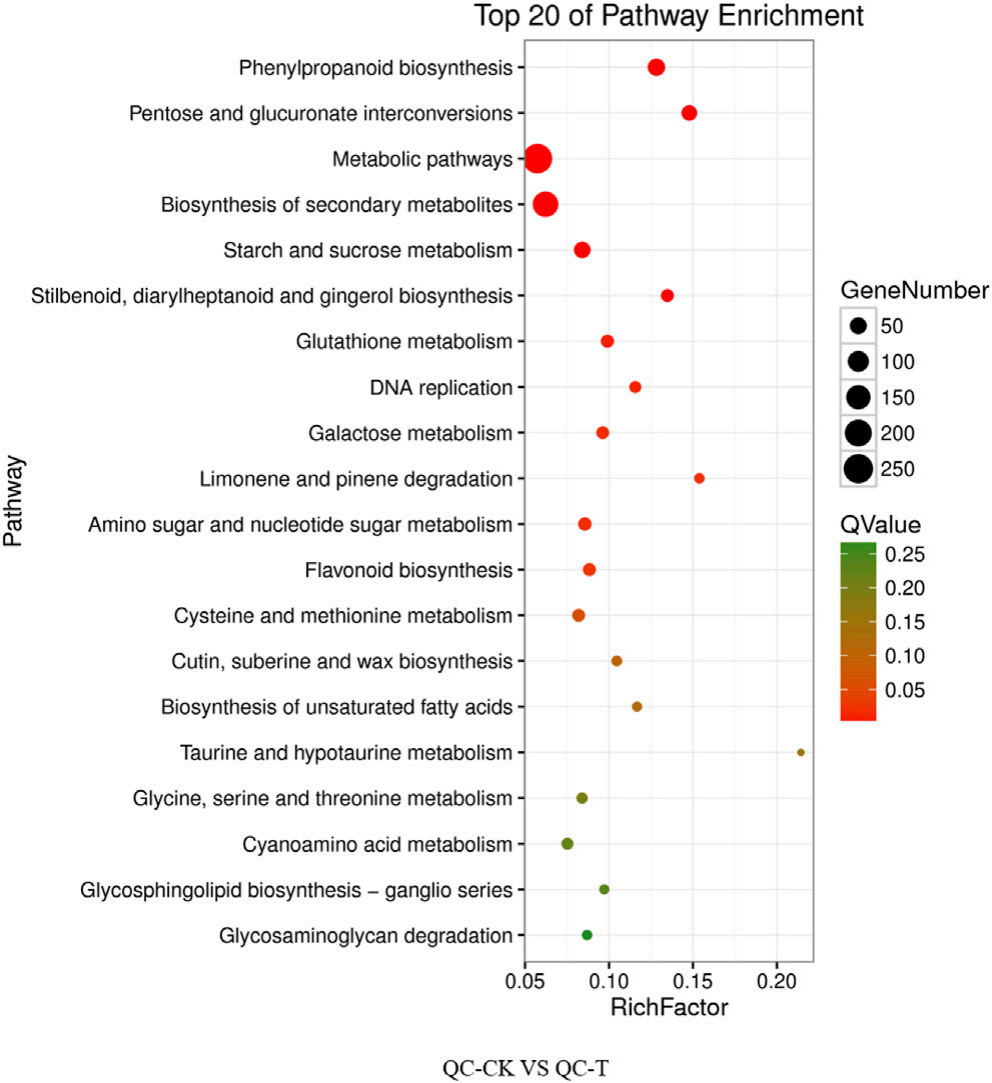
KEGG pathway of cutting tea plants under drought on day 0 and day 20. QC, Cutting tea plant. CK: Cutting samples treated with drought for 0 days, T: Cutting samples treated with drought for 20 days.

**FIGURE 7:**
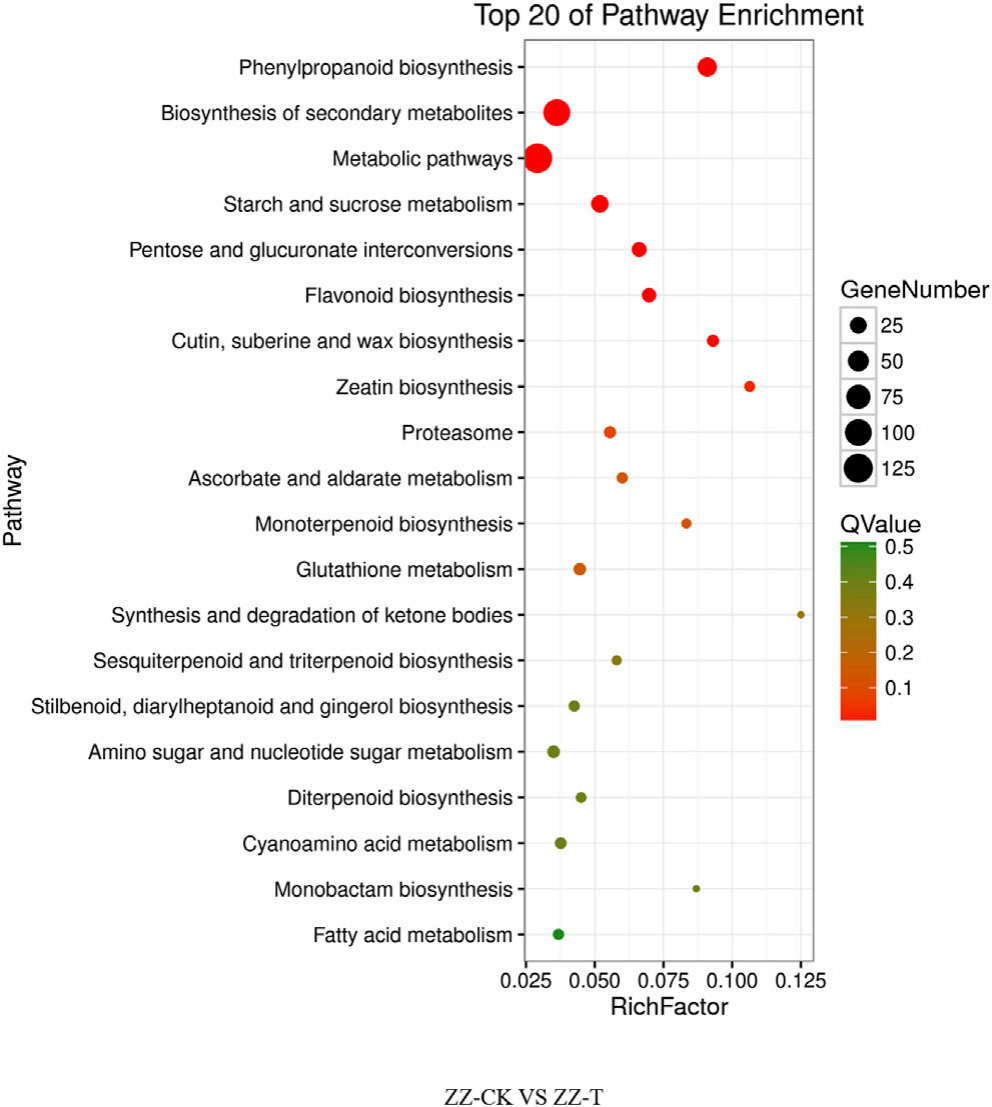
KEGG pathway of seeding tea plants under drought on day 0 and day 20. QC, Cutting tea plant. CK: Cutting samples treated with drought for 0 days, T: Cutting samples treated with drought for 20 days.

The differential genes of KEGG pathway in the offspring of tea cutting propagation are mainly enriched in metabolic pathways such as metabolic pathways, biosynthesis of secondary metals, phenylpropanoid biosynthesis, and so on (Figure 6). The treatment results of the actual offspring of tea seed reproduction can be divided into two categories: the first involves gene expression, such as DNA replication, and the second involves metabolism, such as phenylpropanoid biosynthesis, biosynthesis of secondary metabolites, the metabolic pathways, pentose glucuronate interconversions, starch and sucrose metabolism, flavonoid biosynthesis, zeatin biosynthesis, Cetin, suberine and wax biosynthesis, proteasome, and so on (Figure 7).

### Effects of Moderate Drought on Pro Metabolism-Related Genes in Tea Roots

Among the different genes in the asexual progeny of tea cutting propagation, the genes related to Pro metabolism and significantly down-regulated are as follows:AZT1 (TEA006283.1, azelaic acid inducible 1), THE1 (TEA018952.1, protein kinase family protein), At4g12490 (TEA019367.1, bifunctional inhibitor/lipid transfer protein/seed storage 2S albumin superfamily protein), PROT1 (TEA013759.1, proline transporter 1), and At1g49730 (TEA013092.1, protein kinase superfamily protein). It has been reported that AZI1 and MPK3 interact with each other to form protein complexes in plants, which have certain effects on drought resistance of tea plants. THE1, a malectin-like receptor kinase previously known as a cell wall integrity sensor, is responsible for drought resistance in tea plants, depending on the root tilt and salt stress sensitivity phenotypes. At4g12490 is involved in plant pathogen defense and stress tolerance. Overexpression of this gene will increase root growth of tea plant under drought stress. PROT1 plays an important role in the protective stress response of tea plants, and plays a protective role when tea plants are subjected to drought. The expression of At1g49730 was induced in tea plant during drought and high temperature, which encodes a protein kinase with unknown function. AZT1 was one of the significantly down-regulated genes related to Pro metabolism in the different genes in the offspring of tea seed reproduction.

### Effects of Moderate Drought on Peroxidase Metabolism Related Genes in Tea Root

Among the different genes related to POD metabolism that were significantly down-regulated after drought stress, poxN1 (TEA008472.1, peroxidase N1) was found in the asexual descendants of tea cuttage reproduction. PER64 (TEA018992.1, peroxidase superfamily protein) has been reported to play a certain role in the formation of plant second cell wall, which is related to stress. APX3 (TEA027158.1, ascorbic acid peroxidase 3) may play a major role in tea tree adaptation to long-term high temperature stress and antioxidant protection. PoxN1 is one of the significantly down-regulated genes related to pro metabolism in the genes of the descendants of tea seed reproduction.

### Effects of Moderate Drought on Superoxide Dismutase Metabolism Related Genes in Tea Root

SODCC (TEA023332.1, superoxide dismutase [cu-zn]) has been reported to play an important role in removing active oxygen species in tea plants and is related to drought resistance.

### Validation of Transcriptome Data by qRT-PCR

**FIGURE 8:**
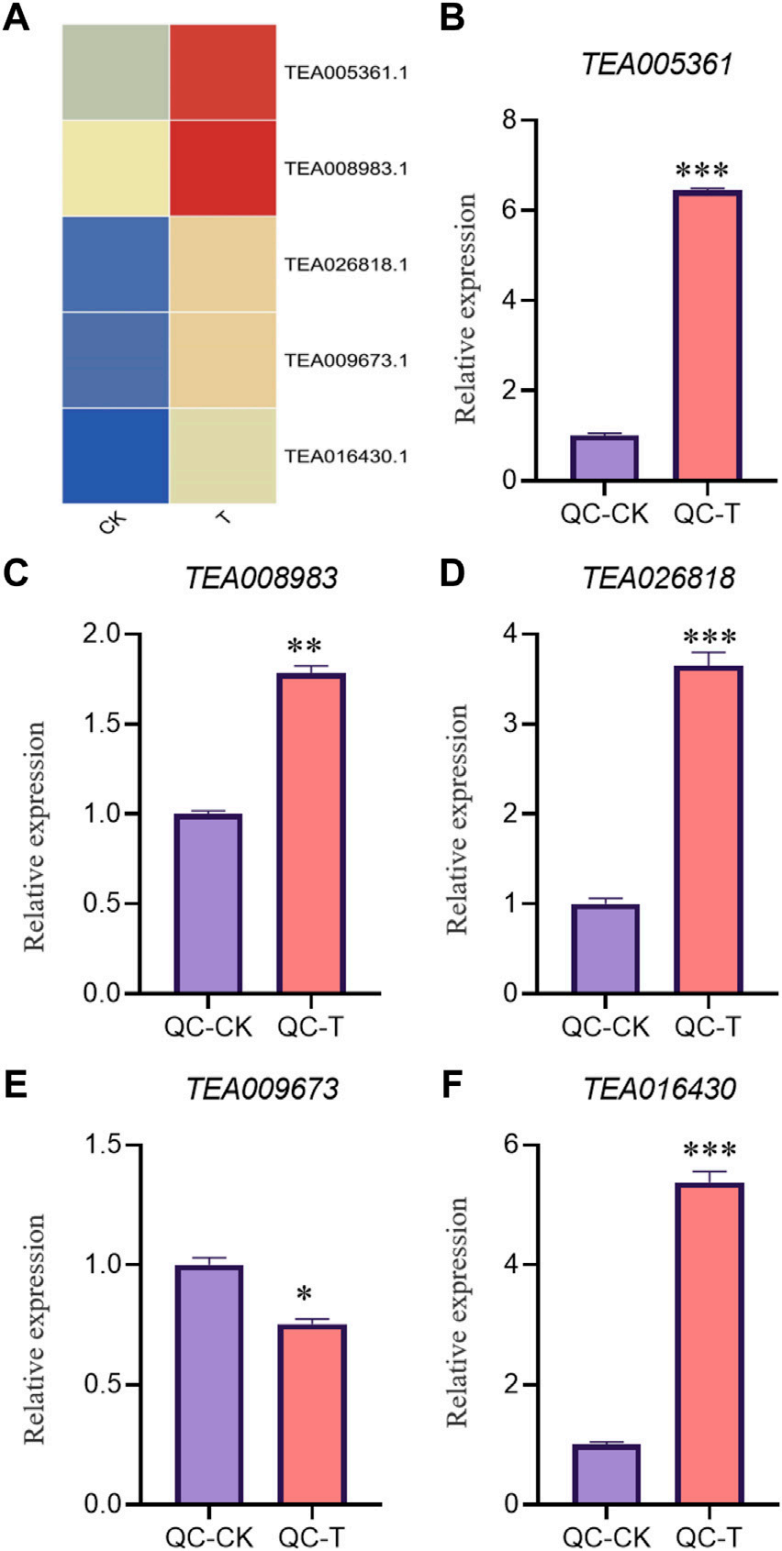
QC-CK&QC-T Comparison of unregulated genes expression pattern between RNA-Seq and qRT-PCR results. The gene expression values were normalized to the Actin gene. The purple column represents the results of drought treatment for 0 days, and the orange red column represents the results of drought treatment for 20 days. CK: Cutting samples treated with drought for 0 days, T: Cutting samples treated with drought for 20 days. **p < 0.01, ***p < 0.0001.

**FIGURE 9:**
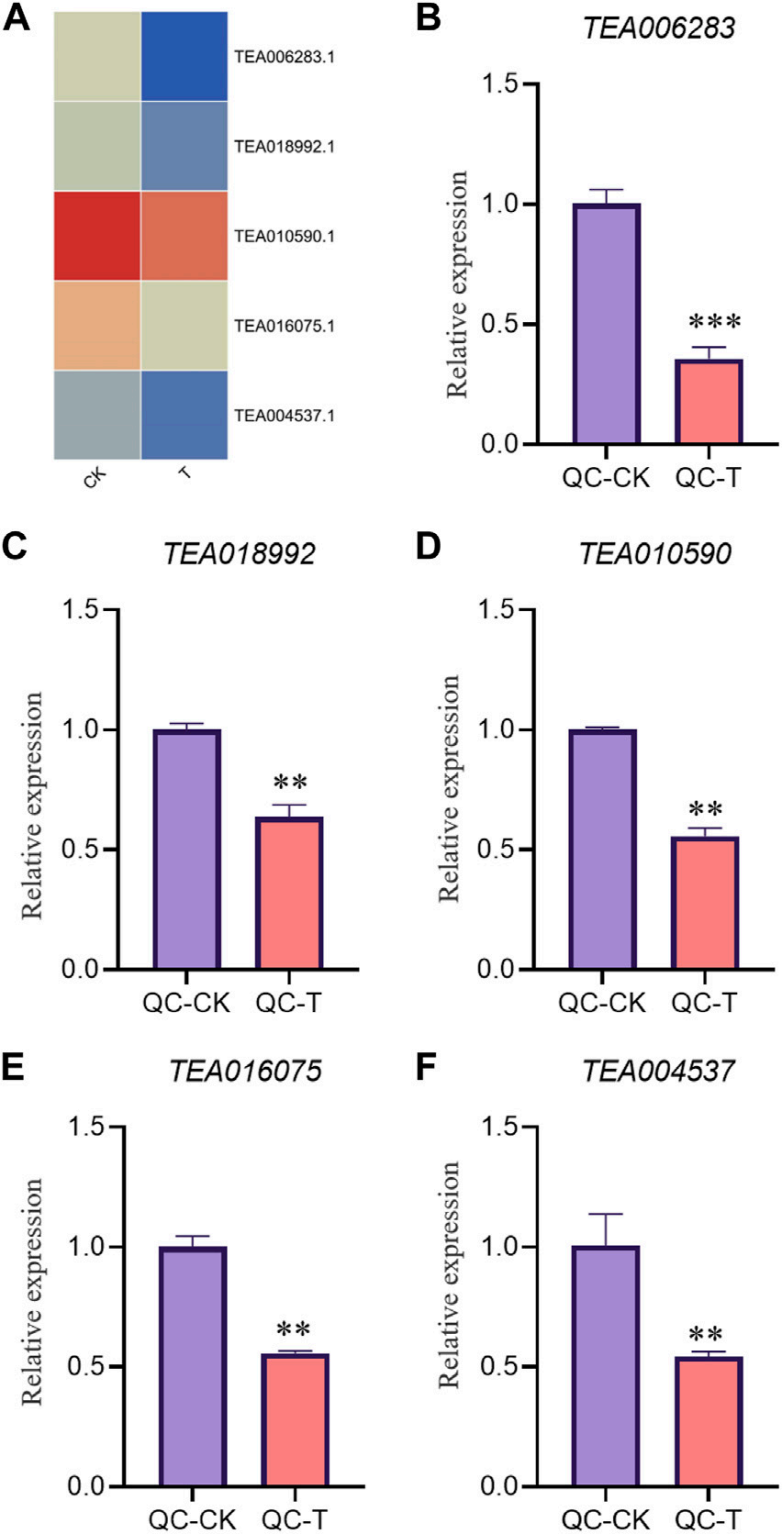
QC-CK&QC-T Comparison of down genes expression pattern between RNA-Seq and qRT-PCR results. The gene expression values were normalized to the Actin gene. The purple column represents the results of drought treatment for 0 days, and the orange red column represents the results of drought treatment for 20 days. CK: Cutting samples treated with drought for 0 days, T: Cutting samples treated with drought for 20 days. **p < 0.01, ***p < 0.0001.

**FIGURE 10:**
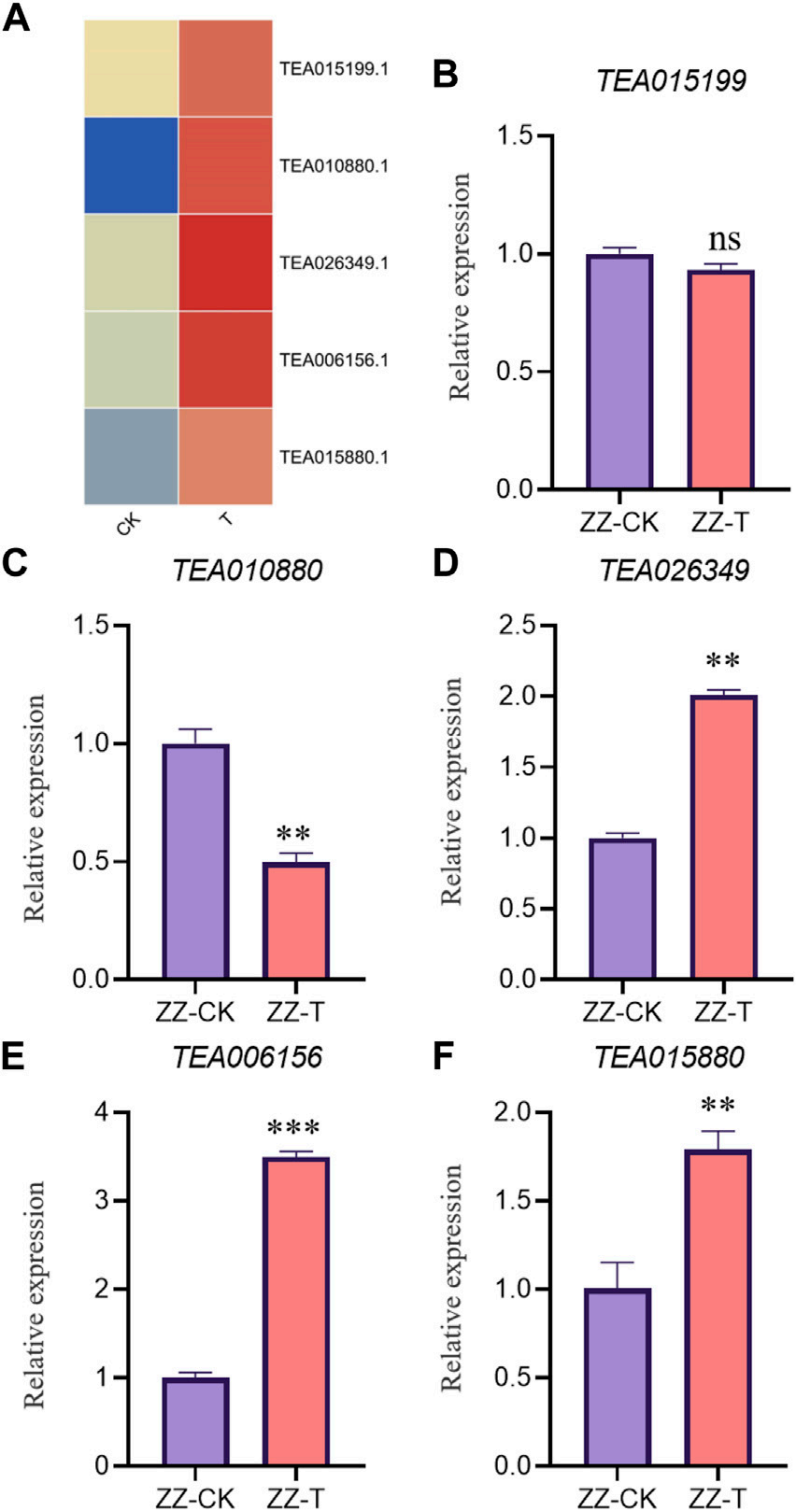
ZZ-CK&ZZ-T Comparison of unregulated genes expression pattern between RNA-Seq and qRT-PCR results. The gene expression values were normalized to the Actin gene. The purple column represents the results of drought treatment for 0 days, and the orange red column represents the results of drought treatment for 20 days. CK: Seeding samples treated with drought for 0 days, T: Seeding samples treated with drought for 20 days. **p < 0.01, ***p < 0.0001.

**FIGURE 11:**
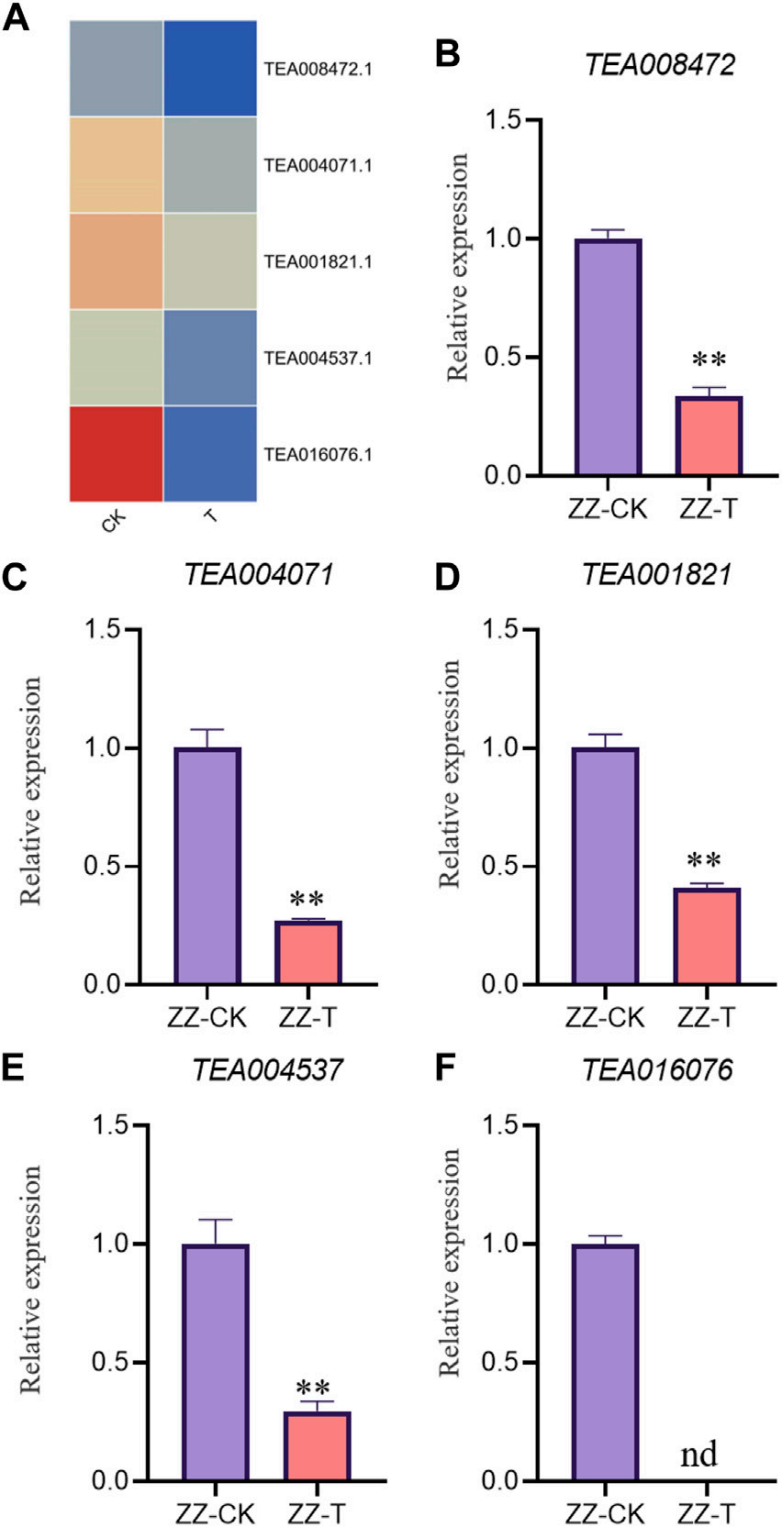
ZZ-CK&ZZ-T Comparison of down genes expression pattern between RNA-Seq and qRT-PCR results. The gene expression values were normalized to the Actin gene. The purple column represents the results of drought treatment for 0 days, and the orange red column represents the results of drought treatment for 20 days. CK: Seeding samples treated with drought for 0 days, T: Seeding samples treated with drought for 20 days. **p < 0.01, ***p < 0.0001.

**FIGURE 12:**
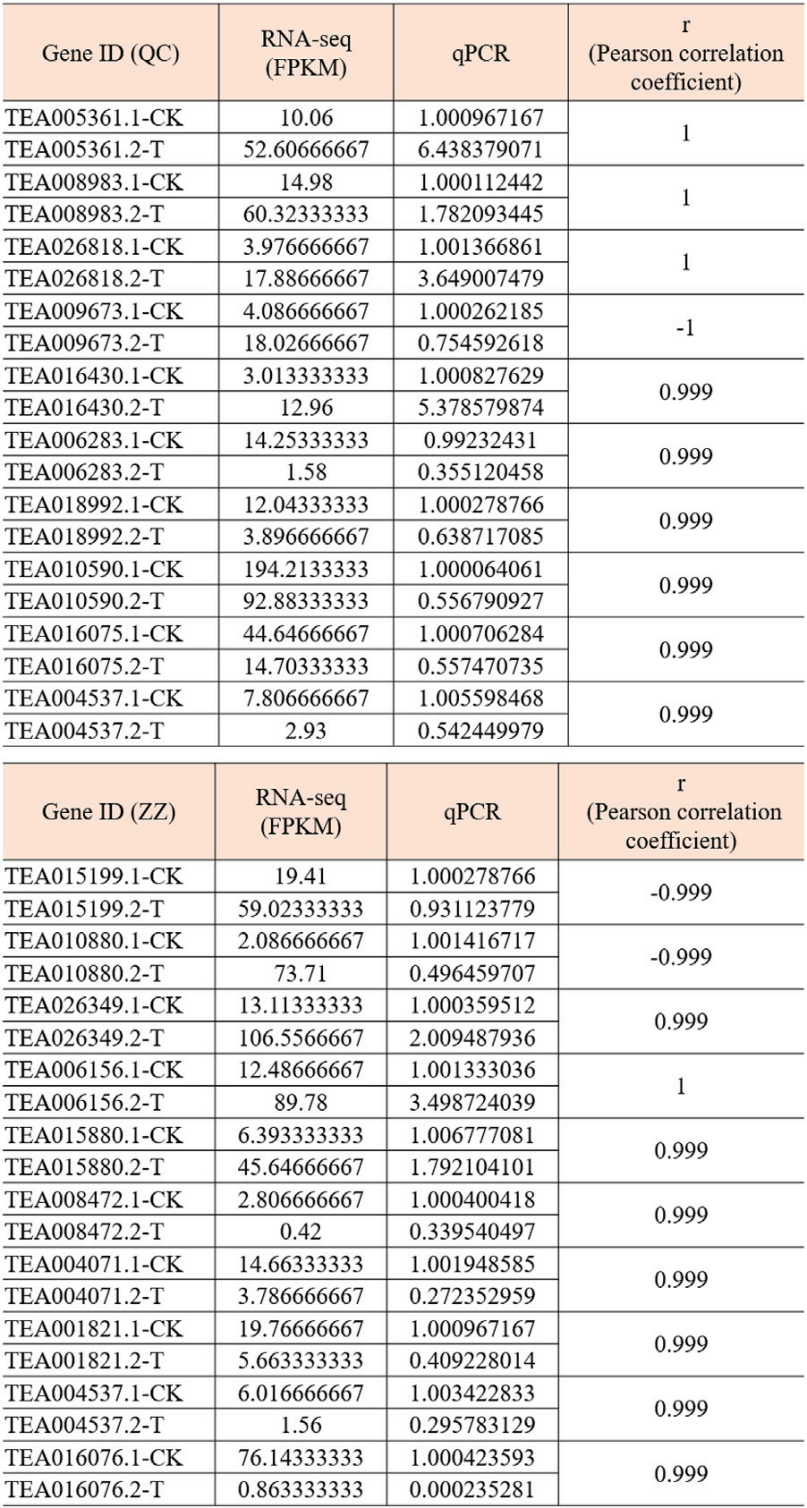
Correlation analysis between RNA-seq and qPCR. Pearson coefficient is used for correlation analysis, and r represents the correlation coefficient.

In order to verify the accuracy of transcriptome, we screened 5 up-regulated genes and 5 down-regulated genes from QC and ZZ, respectively, for fluorescence quantitative PCR verification. The results showed that in QC treatment, except TEA009673, other genes were consistent with transcriptome data, whether up-regulated genes or down-regulated genes (Figures 8, 9). In ZZ processing, TEA01599 and TEA010880 are slightly different from transcriptome data, and other values are highly correlated with transcriptome data (Figures 10, 11). The qRT-PCR analysis suggested that the RNA-seq results were reliable (Figure 12).

## Discussion

Drought resistance is a complex biological process. Multiple genes related to various biochemical and physiological processes of plants interact to dynamically control and regulate drought resistance (Ansari et al., 2022). In this study, the physiological, biochemical, and transcriptome responses of cutting and seed propagation tea plants to drought stress were combined to comprehensively understand the genetic control of drought resistance of tea plants. Previous studies showed that most DEGs tended to increase with the intensification of drought (Li et al., 2020). However, our results showed that the down-regulated genes were significantly higher than the up-regulated genes, which may be caused by the change of regulated genes due to the different cultivars selected, but the specific mechanism still needs further study.

Metabolic regulation is the key mechanism for plants to maintain cell osmotic potential under drought stress. Plant primary and secondary metabolites and metabolic genes participate in a variety of metabolic pathways and are the key factors of plant drought resistance. Physiological, biochemical, and molecular strategies of plant drought resistance mechanism can be used to improve the survival rate of plants under drought stress (Kumar et al., 2021). Studies have shown that aquaporins play a key role in the regulation of plant hydraulic conductivity at the molecular level. They are identified and found in different plant chambers of grapevine and expressed in all tissues of grapevine (Sabir et al., 2021). In this study, GO function analysis showed that the drought raised and lowered the expression of genes in sexual health offspring and asexual reproduction, the distribution of functional parts on the GO, in the biological control, composition organization or biological cells, cell processes, metabolic process, positioning, stimulus-response, single biological process, cells, cell components, the extracellular region, membrane components, organelles composition, bonding, catalytic activity and transport activity significantly enriched in functions, such as that involved in the process of tea plant drought resistance abundant cell reaction.

Relevant studies comprehensively analyzed the global changes of protein and mRNA abundance regulated by fulvic acid in plant leaves under drought stress. It was found that FA enhanced drought resistance by regulating starch and sucrose metabolism, phenylpropane metabolism, triterpene biosynthesis, and HSPs (Qiu et al., 2021). Our transcriptome analysis found that, KEEG pathway enrichment analysis of differentially expressed genes showed that the enrichment pathways of sexual and asexual progeny overlaps, such as phenylpropanin biosynthesis, secondary metabolic biosynthesis, metabolic pathways, starch and sucrose metabolism, pentose and glucuronic acid conversion, and flavonoid biosynthesis. In addition, biological synthesis of keratin and wax, zeaxin biosynthesis, and proteasome are also involved in the enrichment of sexual progeny. The enrichment pathways of asexual progeny include biosynthesis of distyrene diarylheptanoic acid and gingerol, glutathione metabolism, galactose metabolism, degradation of limonene and pinene, amino sugar and nucleotide sugar metabolism. Although most of them are involved in metabolism, there are still differences between the two pathways, indicating that there may be differences between the molecular mechanisms of sexual and asexual offspring of tea plants in coping with drought stress, which needs further verification.

Plants can integrate different stress signals and improve their chances of survival by inducing stomatal responses and the production of reactive oxygen species (ROS) and hormones (Zhao et al., 2022). Oxygen-scavenging system can be used as a descriptor for identifying drought-tolerant accessions (Rahimi et al., 2021). The analysis of transcriptome results also found that moderate drought had a great impact on the expression of Pro-, SOD-, and POD-related genes in tea roots. The genes significantly related to Pro and POD were significantly down-regulated, while the genes significantly related to SOD were up-regulated.

## Conclusion

The above research results revealed that moderate drought stress on different breeding way tea tree root molecular mechanisms of metabolic control, to further study of some of the key functional genes, will help improve tea cultivation way, cultivating high effective constituents’ content and strong drought resistance varieties of tea tree, further improve the yield and quality tea.

## Data Availability

The data presented in the study are deposited in the Sequence Read Archive(SRA) repository, accession number PRJNA822682. The data was released on 2022-05-01.

## Abbreviations

CAT, catalase; DEG, differentially expressed genes; GAPDH, gluceraldehyde phosphate dehydrogenase; GO, Gene Ontology; KEGG, Kyoto Encyclopedia of Genes and Genomes; POD, peroxidase; QC, cuttings of tea plant; qRT-PCR, quantitative real-time PCR; ROS, reactive oxygen species; SOD, superoxide dismutase; ZZ, seeds propagate tea plant.
